# Including Thermal Fluctuations in Actomyosin Stable States Increases the Predicted Force per Motor and Macroscopic Efficiency in Muscle Modelling

**DOI:** 10.1371/journal.pcbi.1005083

**Published:** 2016-09-14

**Authors:** Lorenzo Marcucci, Takumi Washio, Toshio Yanagida

**Affiliations:** 1 Department of Biomedical Sciences, Padova University, Padova, Italy; 2 Quantitative Biology Center, RIKEN, Suita, Japan; 3 Graduate School of Frontier Sciences, The University of Tokyo, Tokyo, Japan; University of California San Diego, UNITED STATES

## Abstract

Muscle contractions are generated by cyclical interactions of myosin heads with actin filaments to form the actomyosin complex. To simulate actomyosin complex stable states, mathematical models usually define an energy landscape with a corresponding number of wells. The jumps between these wells are defined through rate constants. Almost all previous models assign these wells an infinite sharpness by imposing a relatively simple expression for the detailed balance, i.e., the ratio of the rate constants depends exponentially on the sole myosin elastic energy. Physically, this assumption corresponds to neglecting thermal fluctuations in the actomyosin complex stable states. By comparing three mathematical models, we examine the extent to which this hypothesis affects muscle model predictions at the single cross-bridge, single fiber, and organ levels in a *ceteris paribus* analysis. We show that including fluctuations in stable states allows the lever arm of the myosin to easily and dynamically explore all possible minima in the energy landscape, generating several backward and forward jumps between states during the lifetime of the actomyosin complex, whereas the infinitely sharp minima case is characterized by fewer jumps between states. Moreover, the analysis predicts that thermal fluctuations enable a more efficient contraction mechanism, in which a higher force is sustained by fewer attached cross-bridges.

## Author Summary

Mathematical models are of fundamental importance in the quantitative verification of biological hypotheses. Muscle contraction models assume the existence of several stable states for the myosin head, whereas the transition rates between states are defined to fit experimental data. The ratio of the forward and backward rates is linked to the ratio of the probabilities of being in one or other stable state at equilibrium through a detailed balance condition. A commonly used assumption leads to a relatively simple expression for this balance condition that depends only on the values of the energy at the minima and not on the minima shape. Mathematically, this hypothesis corresponds to infinite sharpness at these minima; physically, it neglects the small thermal fluctuations within actomyosin stable states. In this work, we compare this classical approach with a model that includes thermal fluctuations within wide minima, and quantitatively assess how much this hypothesis affects the model outcomes at the single molecule, single fiber, and whole heart levels. It is shown that, using parameters compatible with known behavior in muscle mechanics, relaxing the infinitely sharp minima hypothesis improves the predicted force generation and efficiency at the macroscopic level.

## Introduction

Intracellular forces and motions are generated by cyclic interactions between myosin and actin filaments. Myosin–actin interactions govern many important phenomena, such as molecular transport and muscle contraction. In the currently accepted theory, the globular portion of myosin firmly attaches to the actin filament until an ATP molecule, which fuels the molecular motors, binds to the myosin catalytic domain, releasing the myosin from the actin. The ATP is then hydrolyzed, allowing the myosin to weakly interact with the actin without generating any force. As the interaction strengthens and the hydrolization products are released, the myosin head can generate forces or motions that modify its configuration into one or more relatively stable states. When a new ATP molecule binds to the myosin molecule, it detaches from the actin and the cycle repeats.

The driving force of actin-attached myosin is sourced from its total potential energy *E*_*t*_. The total actomyosin potential energy may be decomposed into two components. The first is generated by the chemical force field acting at the actin and myosin interface during their strong interaction, when the actomyosin complex is formed. We call this the *biochemical* energy *E*_*c*_. The second component is generated by the mechanical force field associated with the myosin protein internal deformation; we call this the *mechanical*, or elastic, energy *E*_*e*_. The balance between these two components defines the relative stability of the stable states of the attached myosin head [1].

**Fig 1:**
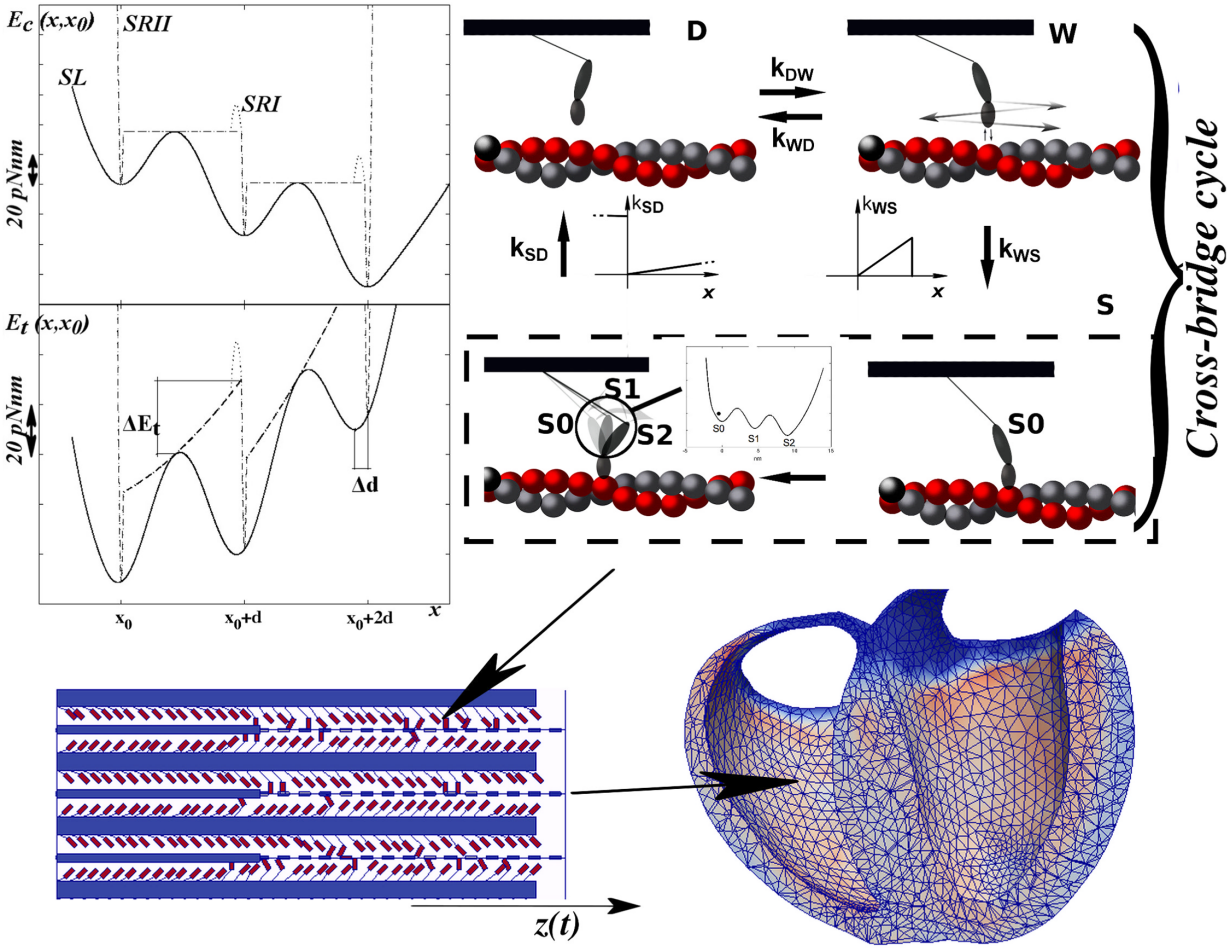
Model description. Top-left: Unloaded or biochemical energy of the actomyosin complex (upper) and total energy given by the sum of the biochemical energy and the elastic parabolic energy due to an elastic element (lower, with *x*_0_ = −2*nm*). SRII (dot-dashed line) and SRI (dotted line, with a higher energetic barrier that makes the backward rate a constant) have sharp minima, whereas SL (continuous line) has wide minima. The lower panel shows the effect of the shape of the biochemical energy on the minima locations and the energetic barrier of the total energy *E*_*t*_(*x*, *x*_0_); the sharper the minima, the more rigid the positions (this effect is related to the mechanical energy). In the wide minima case, the forward (in the direction of motion of the myosin) minimum shifts toward the previous one. In the same way, the energy maximum is reduced in SL (relative to SRI and SRII) because it occurs at a less stretched position of the elastic element. Top-right: Cross bridge cycle. Myosin molecular motors in the detached state (D) can attach weakly (W) to the actin filament, still without generating any force. Myosin from the weak state can detach or attach in the strongly attached state in the pre-power-stroke configuration S0, following a Huxley 1957 (H57) [2] attachment rate function. In the strongly attached state, the myosin head can exist in three different states: one pre-power-stroke and two post-power-stroke states (S1 and S2). In the strongly attached state, myosin can detach from any state, starting its cycle again. Bottom-left: geometry of the single half-sarcomere, formed by NFil actin filaments with Nxb cross-bridges. Under the hypothesis of uniform behavior, the half-sarcomere is assumed to simulate a single fiber. Actin filaments are attached to the Z-line (*z*(*t*)). The thicker portion of the actin filament overlaps with the filaments in the over-compressed situation. Bottom-right: whole heart simulator, with one sarcomere per finite element (for details, see [21]).

Mathematical models of muscle contraction based on single actomyosin interactions have been extensively analyzed in the literature since the pioneering work of Huxley in 1957 [2]. Several experimental studies have been addressed and different basic hypotheses have since been proposed, because different definitions of these energy landscapes lead to different mathematical predictions of the muscle contraction mechanism. Although the general framework is well established, some hypotheses are still under debate. We do not review the basic hypotheses of these different approaches, as this paper focuses on a particular property of the biochemical energy that is overlooked among the great majority of mathematical models, namely the sharpness of the actomyosin potential wells. We will demonstrate the extent to which this property affects the model outcomes. Theoretical treatments of the rate constant dependence on the total free energy shape were extensively studied in the mid-1970s by Hill and co-workers [3–7]. In particular, in [5], the authors explicitly linked the rate constants between attached states with the total reaction free energy surface given by the sum of the *reaction free energy* (our *E*_*c*_) and the *structural free energy* (our *E*_*e*_). In that paper, a graphical description of the flexibility of the attached myosin motor on the total free energy was given as a contour map of the mechanical *x* and chemical *s* variables, referred to as the *reaction coordinates*. Our bidimensional representation in Fig 1 corresponds to the intersection of that energy with the *reaction path* *x*(*s*), the path of the minimum potential energy [5], which explicitly relates the two variables. However, to the best of our knowledge, this is the first time that this property has been quantitatively investigated by introducing thermal fluctuations within the minima and comparison with the commonly used hypothesis of infinitely sharp minima.

*E*_*e*_ is associated with the deformation of the elastic component of the myosin protein, and is described as a convex energy. We refer to muscle myosin II isoforms, although other myosin types can be treated similarly. The elastic energy *E*_*e*_ has been characterized by applying small force or length perturbations to myofibers [1, 8, 9] or to one/several molecules [10–13]. Although the results vary somewhat among experiments, the mechanical energy component can be completely characterized by determining the myosin elastic stiffness. The location of the flexible part of the myosin molecule, which has not been clarified to date, is not required in this analysis.

Two common features of *E*_*c*_ among different models are: (i) non-convexity, which means that two or more minima, corresponding to actomyosin stable states, are present, and (ii) a bias toward the minimum corresponding to the post-power-stroke state (Fig 1, top-left). Cryo-electron microscopy and X-ray crystallography have confirmed at least two stable conformations of the actomyosin complex, each corresponding to a minimum biochemical energy [14–16]. The observed conformations are usually interpreted by the lever arm model, in which rotation of the long light chain domain of the attached myosin is driven by the energy released by ATP hydrolysis (however, see our Conclusions for a different interpretation). Depending on the external conditions, the long light chain acts as a lever, generating tension or motion. Between these two conformational states, researchers have hypothesized that there exists one or more stable states to explain some macroscopic behavior of the muscle fibers [1, 17–20]. Experimental studies of single myosin molecules have suggested the presence of two power-stroke steps [12].

The most common approach to modeling muscle contractions consists of separating the myosin population into different stable states within the cross-bridge cycle and allowing jumps between states, characterized by forward (*k*_*f*_) and backward (*k*_*b*_) rate constants. A detailed balance requires that the ratio of the two constants is equal to the ratio of the probability distributions of the two states at equilibrium. Each probability distribution is a function of the stretch in the myosin elastic element at the moment of the state transition. Almost all previous models have assumed that this function has a relatively simple exponential dependence of the form *k*_*f*_/*k*_*b*_ = exp (*β*(−Δ*E*_*e*_)) exp (*β*(−Δ*E*_*c*_)), where Δ*E* is the difference between the two energies in the local minima, and *β* = 1/(*κbT*) or *β* = (*κbT*)^−1^, where *κ*_*b*_ is the Boltzmann constant and *T* denotes the absolute temperature. As shown in the Material and Methods section, this equation is only valid under the hypothesis of infinitely sharp minima in *E*_*c*_. This simplification was also used by Hill and coworkers in the quantitative application of Hill’s theoretical formalism to muscle models [3, 6, 7] as well as on later applications of their formalism to different muscle behavior (see for instance [22] and [23]). Although they considered a finite width *within* stable states, they did not include the effects of the width *between* stable states. In other words, they used the simplification that the mechanical coordinate *x* was independent of the reaction coordinate *s*. This hypothesis has sometimes been made explicit, as in the seminal paper of Huxley and Simmons [1] or in the work of Smith and Sleep [24], but is more often assumed implicitly. This approach collapses all the information on the biochemical energy landscape between the minima to a single constant value (the energy barrier), assuming the effect of its shape is negligible with respect to other biochemical energy parameters such as the number of stable minima *n*, the distance between the minima *d*, and the energy drop Δ*E*_*c*_ caused by the ATP bias (see SI).

Physically, the infinitely sharp minimum hypothesis corresponds to a fixed angle of the lever arm in each stable configuration. Thus, thermal fluctuations within the stable states are neglected in the majority of mathematical models, in the sense that they only formally allow for the state transitions, but their effects are collapsed into a single constant value (the unloaded rate). Thermal fluctuations within stable states can be simulated by defining an actomyosin energy landscape with stable minima of finite curvature (Fig 1, top-left). In this paper, we analyze whether and to what extent the width of the actomyosin energy minima in mathematical models may affect the predicted muscle mechanics at three levels: single myosin molecules, single fibers, and macroscopic muscle. To this end, we compare three shapes of the actomyosin biochemical energy in a *ceteris paribus* approach. Maintaining constant values for the actomyosin energy and other parameters characterizing the actomyosin cycle, we find that changing only the width of the energy minima alters the predicted dynamics of the myosin head in the attached state. If the minima are wide, the myosin lever arm can more easily modify its state, moving within the energy landscape. This micro-behavior affects the behavior of single fibers, causing a lower number of attached cross-bridges to generate higher tension in the fiber. Finally, to analyze whether such a difference is preserved at the macroscopic scale, where the huge number of cycling cross-bridges may average out the effects, we apply three models in a finite-element ventricle model [21]. We show that this behavior is preserved at the macroscopic scale of heart contraction, and improves the blood pumping performance. As the minima widen, the predicted ejection fraction increases, relaxation becomes faster, and the heart’s efficiency improves significantly.

Although our analysis can be applied to all myosin isoforms, the mechanism is probably more important for cooperative motors such as muscle myosin. In analyzing the problem from single molecules to macroscopic muscle, we focus on the human cardiac isoform, but our goal is to generalize the relationship between the shape of the biochemical component of the actomyosin energy and the actomyosin dynamics. Hence, to fit the experimental data at each level, we select parameter values that replicate the physiological behavior of the heartbeat, but compare the single-fiber simulations with experimental data from frog skeletal muscle, as these have been intensively reported in the literature (unlike cardiac myosin data).

## Materials and Methods

This paper explores the influence of the actomyosin energy shape on the muscle contraction from the single cross-bridge level to the whole organ by means of mathematical simulations. In the following, we describe the hypotheses and techniques used to simulate the muscle contraction at each level.

### Single molecule

Each myosin head follows the cross-bridge cycle shown in Fig 1 (top-right), with one detached state D, one weakly attached, non-force-generating state W, and an attached state divided in a pre-power stroke S0 and two post-power stroke states S1 and S2 (two-step power stroke). We use the common simplification that one single parameter *x* can describe the position of the myosin head with respect to its relaxed position on the thick backbone at *x* = 0, in a model where the myosin protein is attached to the thick filament through an elastic element of stiffness *k*. We specify that the first minimum (pre-power stroke) of *E*_*c*_ is at *x*_0_(*t*_*a*_), the stretch in the myosin elastic element at the moment of attachment *t*_*a*_. At any time *t* during the actomyosin complex lifetime, we can compute the stretch in the pre-power stroke state *x*_0_(*t*) as a function of the relative sliding of thin and thick filaments *z*(*t*) through *x*_0_(*t*) = *x*(*t*_*a*_) + *z*(*t*) − *z*(*t*_*a*_). *kx*_0_ is the tension of the elastic element in the pre-power stroke state, and is then a function of *z*(*t*). The elastic element is characterized by an asymmetric elasticity [10] with stiffness *k*^+^ = 2 *pN*/*nm* for *x* > 0 (numerical values are listed in S1 Table). Let us consider for the moment only the attached states. We first explicitly derive the dependence of the detailed balance condition from the two components of the total energy, *E*_*c*_ and *E*_*e*_. We can write the total energy of each myosin in *x* as $E_{t}\left(x,x_{0}\right)=E_{c}\left(x,x_{0}\right)+\frac{1}{2}kx^{2}$. The stationary distribution of the probability density then becomes *p*_*s*_(*x*, *x*_0_) = *Nexp*(−*βE*_*t*_(*x*, *x*_0_)), where *N* is the normalization constant. For the sake of simplicity, assume that *E*_*c*_ has only two minima, at *x* = *x*_0_ and *x* = *x*_0_ + *d*, where *d* is the power stroke step, and a maximum in *b* (the reasoning can be extended to the actual case used in this paper, with three minima). At the stationary state, we can define the probability of being in the first minimum as:

$$\pi_{1}\left(x_{0}\right)=\int_{-\infty }^{b}p_{s}\left(x,x_{0}\right)dx$$ (1)

and that of being in the second minimum as:

$$\pi_{2}\left(x_{0}\right)=\int_{b}^{\infty }p_{s}\left(x,x_{0}\right)dx$$ (2)

The detailed balance condition requires that *k*_*f*_/*k*_*b*_ = *π*_2_/*π*_1_. The previous equation cannot be solved without an analytic expression for *E*_*c*_, so *k*_*f*_ and *k*_*b*_, and their ratio cannot be calculated. A very common hypothesis used in almost all mathematical models of muscle mechanics is that the minima in the chemical energy are infinitely sharp, so the exponential of −*βE*_*c*_(*x*, *x*_0_) can be approximated by a delta function whose integral has a non-zero value only at the minima and is zero elsewhere. Under this assumption, the integrals in the detailed balance condition can be explicitly solved leading to *k*_*f*_/*k*_*b*_ = exp(−*β*(*E*_*t*_(*x*_0_ + *d*, *x*_0_) − *E*_*t*_(*x*_0_, *x*_0_))), which can be factorized into the two separate components of the energy. Any model (e.g., [1, 18, 24–27]) that uses such a simplified dependence of the detailed balance on the strain of the elastic element in the pre-power stroke state (*x*_0_) is based on such a hypothesis. In this paper, we develop the theoretical formalism introduced by Hill [5] to account for the thermal fluctuations during the transition between attached stable states, and explore their influence on the model predictions.

We consider a two-step power stroke corresponding to three minima in the biochemical energy. Each step of the power stroke (i.e., the distance between the *E*_*c*_ minima) is *d* = 4.5 *nm*. To appreciate the effect of infinitely sharp minima on the model outcome, we construct three scenarios with different actomyosin energy shapes. Two scenarios (SRI and SRII) are characterized by infinitely sharp minima, but with two different dependencies on the elastic tension. In the third scenario (SL), all of the minima have finite curvature (wide minima). SRI is the classical Huxley and Simmons model [1], in which the backward rates *k*_10_ and *k*_21_ are assumed to be constant and independent of the tension in the elastic element. The forward rates *k*_01_ and *k*_12_ are then defined so that their ratios to the backward rates satisfy the detailed balance. As noted in [24], SRI corresponds to a flat potential energy with a large repulsive energy barrier protecting the post-power stroke minima at *x*_0_ < 0 (Fig 1). In SRII, the minima are again infinitely sharp, but the rate constants are computed through the flat potential energy hypothesis by applying the Kramers–Smoluchovski (KS) approximation:

$$k_{ij}\left(x_{0}\right)={\left[\beta \eta \int_{max_{l}}^{max_{r}}e^{\left(\beta (-E_{t}\left(x,x_{0}\right))\right)}dx\int_{min_{i}}^{min_{j}}e^{\left(\beta (E_{t}\left(x,x_{0}\right))\right)}dx\right]}^{-1}$$ (3)

The first integral is computed between the left and right maxima (*max*_*l*_ and *max*_*r*_) around minimum *i*; the second is computed between minimum *i* and minimum *j* (*min*_*i*_ and *min*_*j*_); for a detailed description, see [28]. As described before, in the infinitely sharp minimum hypothesis, the variation of Δ*E*_*e*_ inside the minimum can be ignored in the first integral, leading to the approximate solution:

$$\int_{max_{l}}^{max_{r}}e^{\left(\beta (-E_{t}\left(x,x_{0}\right))\right)}dx\approx k_{0}e^{-\beta (kd\left(x_{0}+d/2\right))}$$ (4)

where *k*_0_ is a constant related to the reaction rate in the unloaded case. The second integral of Eq (3) is analytically solved in [24].

Scenarios SRI and SRII require constants *k*_*b*_ and *k*_0_, respectively, which are associated with the change of state in the unloaded myosin. These constant values are defined after the description of the third scenario. In the third scenario (SL), the central region of the biochemical energy landscape is defined as:

$$E_{c}\left(x,x_{0}\right)=H\sin\left(2\pi \left(x-x_{0}\right)/d+\alpha_{d}\right)+F_{ATP}\left(x-x_{0}\right)$$ (5)

where the last term simulates the (linear) bias of the biochemical energy generated by the ATP energy release, such that $E_{c}^{S0}>E_{c}^{S1}>E_{c}^{S2}$. *α*_*d*_ is a constant angle that fixes the first minimum in *x* = *x*_0_. The energy barrier in the sinusoidal function is chosen as *H* = 6*κ*_*b*_ *T*_0_ at *T*_0_ = 310.15*K*. We can then approximate the minimum in *E*_*c*_ as a parabola with stiffness *k*_*c*_ = *H*(2*π*/*d*)^2^ = 48 *pN*/*nm*. In this scenario, the detailed balance condition would require a numerical integration at each value of *x*_0_. Instead, the more detailed shape of *E*_*c*_ allows for a different approach. In the numerical simulations of SL, each myosin head was modeled as a material point in over-damped dynamics, experiencing viscous resistance through its drag coefficient *η* = 70 *pNns*/*nm* [29]. This leads to a system of Langevin equations of motion, given by:

$$\eta {\dot{x}}^{i}=-\omega_{i}\left(t\right)E_{c}^{\prime }\left(x^{i},x_{0}^{i}\right)-E_{e}^{\prime }\left(x^{i}\right)+\sqrt{\eta \kappa_{b}T}\Gamma \left(t\right)$$ (6)

for the *i*−*th* myosin. *ω* assumes a value of one or zero when the myosin is in the attached or detached state, respectively, and Γ(*t*) is a random term characterized as white noise by <Γ(*t*)> = 0 and <Γ(*t*_1_)Γ(*t*_2_)> = 2*δ*(*t*_1_ − *t*_2_). The equations are coupled through the dependence of $x_{0}^{i}$ on the relative position of thin and thick filaments *z*. This system was solved by an implicit method (see SI) that exploits the physical properties of the components acting on the myosin dynamics without requiring rate constants. The approach is inapplicable to scenarios SRI and SRII, because the time step is related to the characteristic time of the problem, which is inversely proportional to the sharpness of the minima. In these models, we compute the rate constants at different *x*_0_ using the formulas proposed in [1] for SRI and in [24] for SRII, as described previously. As mentioned earlier, SRI and SRII require two unloaded rate constants (one for each power stroke minimum). To maintain homogeneity when comparing the scenarios, we define the constants such that the unloaded rates in SRI and SRII match the KS approximation of the unloaded energy landscape in SL. In each scenario, the positions of all single molecules were tracked through the numerical simulations, and the total tension was obtained by summing the tensions in the elastic elements of the attached myosin heads.

Finally, to complete the cross-bridge cycle, we must describe the transitions among the strongly attached states and the detached (D) and weakly attached (W) states. The W–S and S–D transitions follow the classical H57 hypothesis [2]; that is, the attachment rate is non-zero only in the stretched configuration of the elastic element (*x* > 0), where it increases linearly with *x* up to *x*_*lim*_. The detachment rate, *k*_*SD*_ in Fig 1, also increases linearly with *x* > 0, and attains a very high value when *x* < 0. This hypothesis, related to the Brownian search and catch mechanism, has been observed in Myosin V [30], and has been extensively used in muscle modeling under different modifications. If the absolute value of *x* exceeds 15 *nm*, mechanical dislodging occurs. The transition from D to W and vice-versa is governed by two rate constants, *k*_*DW*_ and *k*_*WD*_ in Fig 1, which are independent of the tension in the elastic element, but do depend on the calcium concentration and on the sarcomere geometry, as described in the following.

### Single sarcomere

To extend the comparison of the different hypotheses beyond a single molecule, we include them in a bidimensional sarcomere geometry, enabling a quantitative simulation of several experimental protocols. We then use these simulations to compare the different outcomes of the three models in a *ceteris paribus* approach to investigate the consequences of accounting for thermal fluctuations within each stable state. The single half-sarcomere depicted in Fig 1 (bottom-left) is based on the real geometry used. Each thick myosin filament has *N*_*XB*_ myosin heads attached to it, and these can interact with actin filaments. The half-sarcomere is composed of *N*_*Fil*_ parallel thick filaments with an actin filament interposed. Actin filaments are constrained to have the same value of *z* through the Z-line. *z* = 0 refers to the optimal length of the sarcomere. Both filaments are rigid. The actin filaments have troponin-tropomyosin units attached, which tune the D–W interaction according to the Ca concentration (this effect is more important in the heart level model, because the single sarcomere model is always tetanized in the presence of a high Ca concentration). The D–W interaction is also regulated by the cooperativity effect (an increase in actin affinity for myosin with attached neighbor heads) and thin filaments overlapping due to over-compression. The three mechanisms are described in SI, and in [21]. Using the parameters described above (which are identical in the three actomyosin energy scenarios), we simulate the tension generated in the filaments under various experimental protocols, namely isometric contraction, isotonic shortening, and the length-step protocol [1].

Most parameters remained unchanged throughout the simulations reported in this paper. The exception is the temperature, which (to match the experimental data) was set to 37°C in the beating heart analysis and to 4°C in the single-molecule and single-fiber analyses. The temperature effect was modeled through the *Q*_10_ parameter in the attachment and detachment rate constants [31], and was intrinsically included in the KS approximation of the power stroke rate constants.

### Heart

To test the influence of the different hypotheses when thousands of myosin heads are working in unison under physiological conditions, we coupled the sarcomere models described in the previous subsection with the finite-element ventricle model, a complete description of which is given in [21]. In each finite element, we included the tension generated by the three different sarcomere models, scaled over the area of each finite element. In doing so, we made the assumption that the single half-sarcomere is representative of the tension generated by the whole muscle cell (homogeneity of contraction). The numerically simulated Langevin approach is unsuitable for a heart simulator. Even in the implicit scheme, the characteristic time scales of the model limit the maximum time step to a few nanoseconds. To simulate several heart beats with thousands of elements, we used the KS approximation of the wide minima scenario (see Eq (3)). The two forward and two backward rates, *k*_01_, *k*_10_, *k*_12_, and *k*_21_, were computed at 10^2^ discrete intervals of the stretching of the elastic component in the interval −20 *nm* ≤ *x*_0_ ≤ 20 *nm*. Moreover, in both the detached and attached states within each minima, the position of each myosin head was randomly selected from the corresponding probability distribution (see SI), reproducing the effect of thermal fluctuations on the actual stretching level of the elastic element. To test the new approach, we compared it with the SL and SRII scenarios at the single cross-bridge and whole-fiber levels. The approximation agreed with the Langevin approach (see S3 and S4 Figs), especially in the comparison with SRII. In the numerical experiments, the ventricle wall was discretized into 45,256 tetrahedral elements (Fig 1, bottom-right). The macroscopic contraction force along the prescribed fiber orientation in each element was provided by 12 embedded filament pair models. The macroscopic contraction force was specified such that the sum of the work done by each actomyosin complex equaled the work done by the contraction during an infinitesimal deformation of the continuum. The shortening velocity of the sarcomere was *dz*/*dt* = −(*SL*_0_/2) × *d*λ/*dt*, where *SL*_0_/2 is the initial half-sarcomere length and λ is the magnitude of stretch along the fiber orientation. With this construction, we could explore, at the organ level, the physiological meanings and impacts of different energy landscapes in the three scenarios.

## Results and Discussion

Under the above-described hypotheses, three single-molecule models were developed and incorporated into a single-sarcomere model. Finally, the sarcomere model was integrated into a finite-element ventricle model. Only the sharpness of the minima were modified, which can be physically interpreted as allowing or forbidding thermal fluctuations in the stable states of the actomyosin complex. This thermal property, which is usually neglected in mathematical models of muscle mechanics, was found to fundamentally change the predicted behavior at the microscopic (actomyosin complex), mesoscopic (single fiber), and macroscopic (heart) scales.

### Single molecule

**Fig 2:**
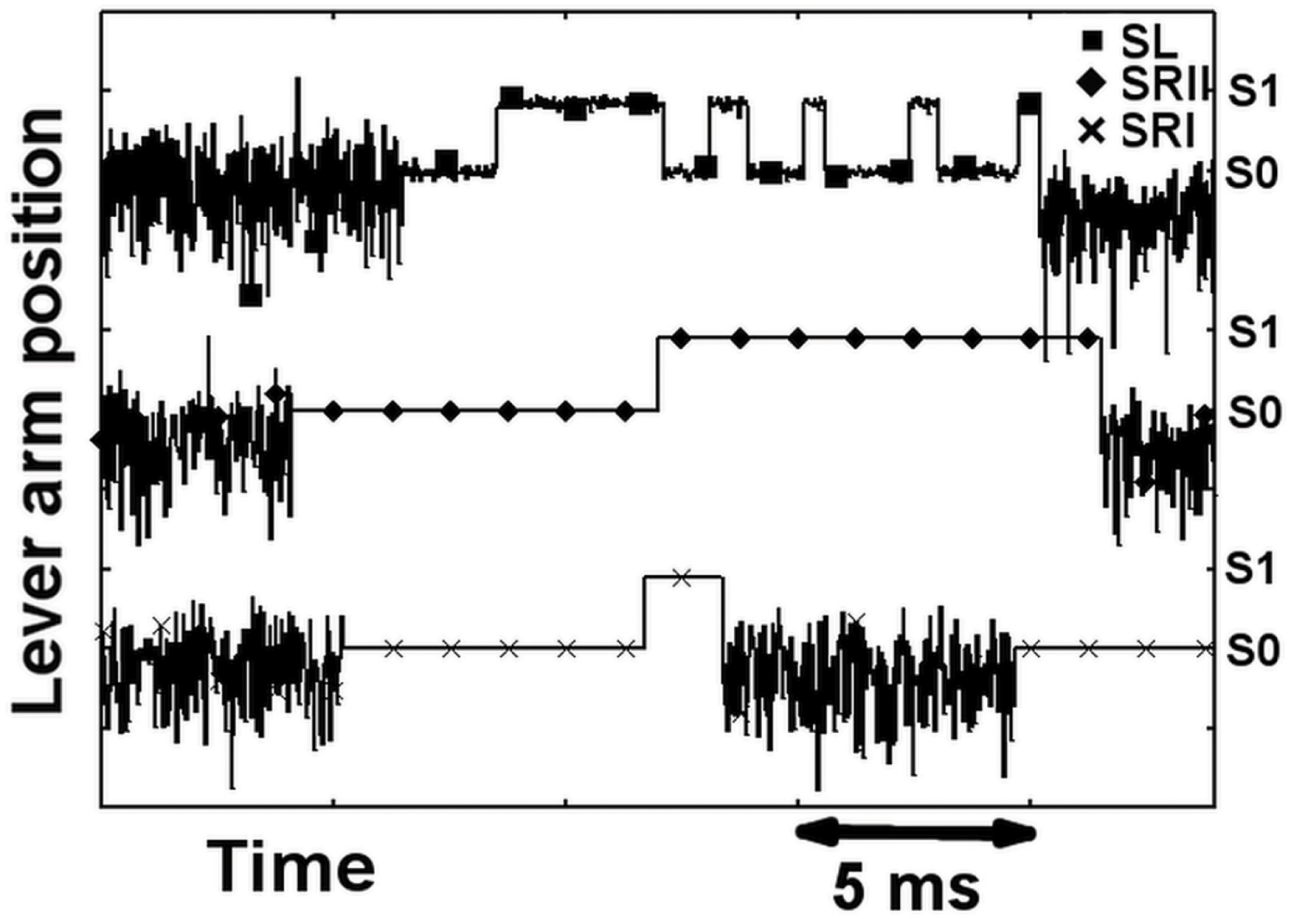
Dynamic behavior of the cross bridges in time. In each scenario the myosin head freely fluctuates against the sole myosin stiffness in the D or W state. The attachment event is followed by an abrupt decrease in the variance of the position of the lever arm. In the SL scenario, the lever arm exhibits small fluctuations within the stable attached states, whereas in the sharp minima cases, they are completely prevented. The traces refer to the isometric contraction, where the lever arm populates its stable positions S0 and S1, but S2 is almost prevented by the high mechanical energy required.

The first effect of including thermal fluctuations in the actomyosin complex is directly appreciated at the single-molecule level. Recall that the power-stroke parameters are the same in all three scenarios, and that each scenario differs only in the shape of the minima in the energy landscape. The number and separation distance of the minima, myosin stiffness, and drag coefficient are the same. As previously described, we also set the rate constants of the unloaded myosin to be the same in all three cases. This is equivalent to imposing the same energy barriers between the minima. Nonetheless, the dynamics of the actomyosin complex during the same attachment–detachment event differed substantially among the scenarios. Fig 2 shows representative traces in the three scenarios. The simulations reproduce an isometric contraction (*z*(*t*) = 0). After attachment in the pre-power stroke state, the lever arm must overcome an energy barrier to generate the power stroke. This barrier depends on both the biochemical and mechanical parts of the actomyosin energy. In SL, the actomyosin state changes several times during the actomyosin lifetime, even in an isometric contraction. In contrast, the SRI and SRII scenarios generate only one or a few power stroke events. With infinitely sharp minima, the power stroke exhibits an on–off behavior. The myosin attaches in the pre-power stroke state, generating low or zero force, and then undergoes a single transition to a completely different state. This new state generates a higher force, pulling the thin filament as far as possible before one ATP arrives. The ATP detaches the head and reprimes the lever arm of the myosin. The wider minima generate a more dynamic framework. In this case, the power stroke is no longer a single event; instead, the actomyosin complex exists as a family of states. After attaching in the pre-power stroke, the myosin head can easily explore the energy landscape before or after the release of the hydrolization products. In some sense, the wider minima case corresponds to a loose coupling of the mechanical event and release of the ATP hydrolization products. The lever arm can more easily move between stable states in the presence or absence of Pi and ADP [32]. In contrast, the sharp minima are more closely related to tight coupling, with one (or few) transition(s) per attachment–detachment event.

This different behavior can be explained by superposing the biochemical and mechanical energy landscapes of the moving particle in the SL and SR scenarios (Fig 1, top-left). With the parabolic approximation described above, we can compute the shift of each minimum Δ*d* (shown in Fig 1, top-left) as a function of *k*_*c*_ and *x*_0_:

$$\delta d=(1-\frac{1}{k/k_{c}+1})(\bar{x_{0}})$$ (7)

with ${\bar{x}}_{0}=x_{0}+nd$, where *n* = 0, 1, 2 for S0, S1, or S2, respectively. The power stroke step then depends on the tension of the elastic element in SL, whereas in SRII (or SRI) with *k*_*c*_ = ∞, the power stroke step has a constant distance *d*, and Δ*d* = 0. A similar effect applies to the maximum in the energy barrier, which is shifted toward a position of lower mechanical energy for wider minima, reducing the energetic barrier (Fig 1). Thus, under the wider minimum hypothesis, the dynamics of the myosin head cause the transition rate to increase at higher tensions, despite being the same in the unloaded condition.

Obviously, the differences between the scenarios are related to the relative rates of the attachment–detachment events and the power stroke event. Nonetheless, with the parameter values used to generate Fig 2, we can quantitatively fit the microscopic model to the macroscopic experimental observations, as shown in the following subsections.

### Single sarcomere

**Fig 3:**
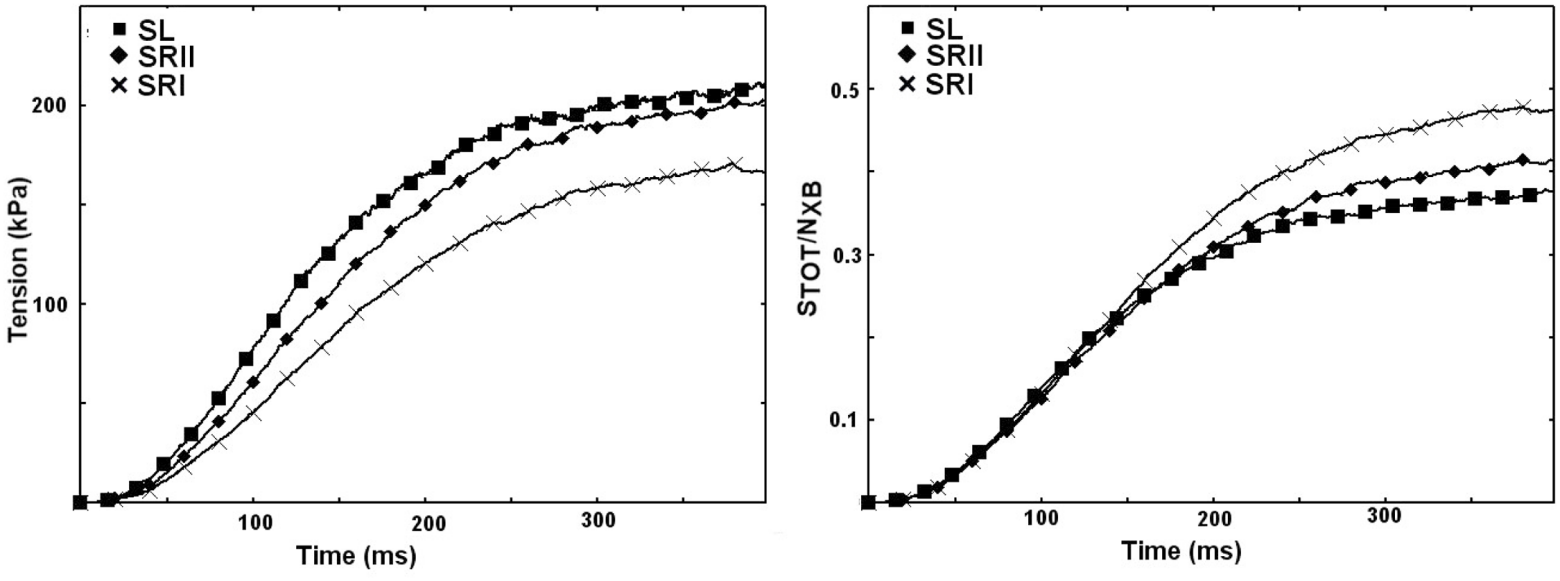
Faster state transitions in SL than in the other scenarios generate a more populated S1 state in the rising phase. Consequently, the total tension is higher in SL than in SRI and SRII (left panel). Moreover, the H57 hypothesis on the detachment rate generates fewer attached cross-bridges (right panel, *S*_*TOT*_ is the sum of myosin in each of the attached states S). In this way, the wider minima optimize the work done by each cross-bridge, and the average force in the cross-bridges is higher than in the SR cases.

**Fig 4:**
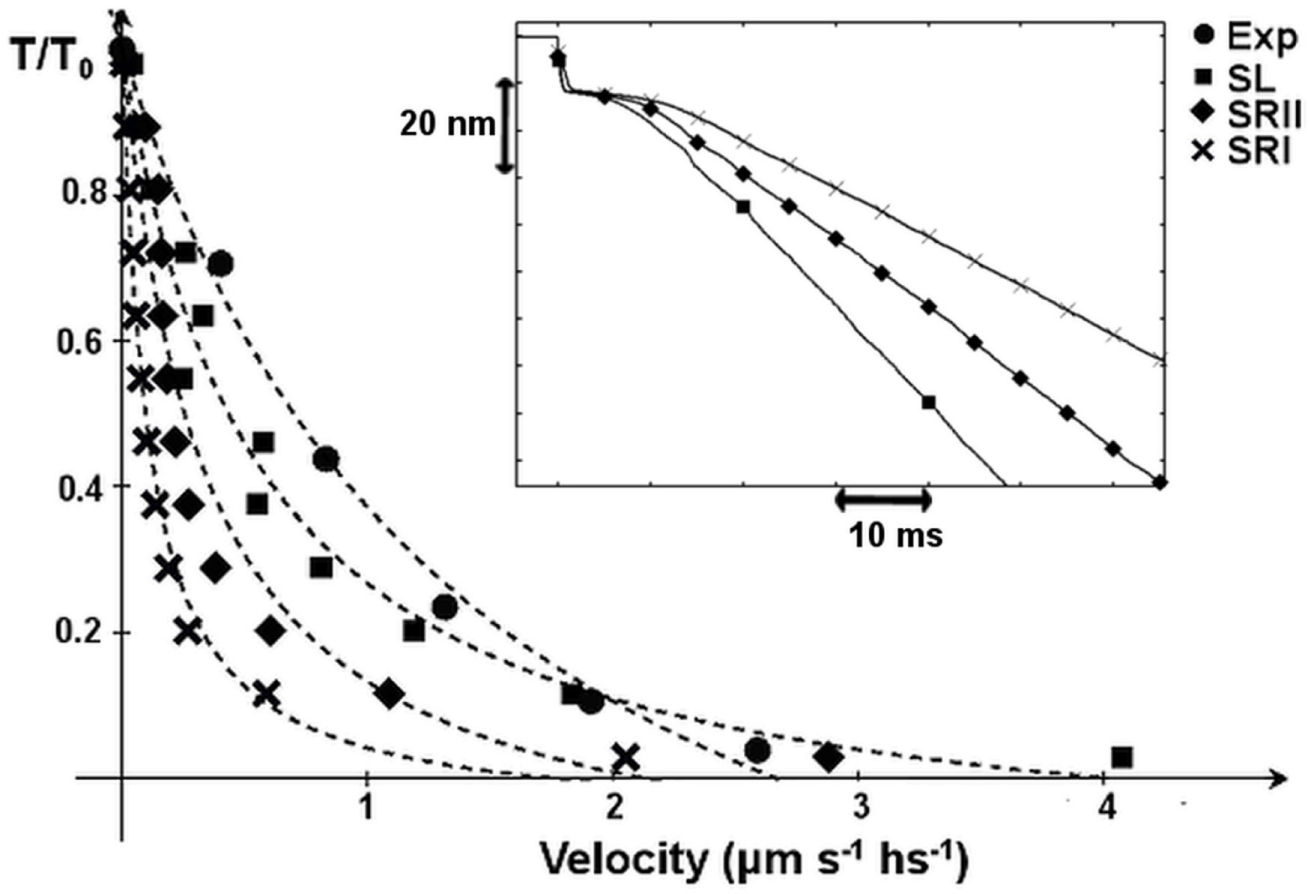
Isotonic contraction experiments give the force velocity curve (dashed lines represent the Hill’s curve fitting). Experimental data are from [33]. The SL model produces a higher contraction velocity under the same tension with respect to SRII and SRI, where the latter is the slower case. The boxed inset shows an isotonic experiment at *T* = 0.2*T*_0_. All scenarios qualitatively reproduce the four experimentally observed phases [34].

As mentioned above, widening the minima shifts the minima of the post-power strokes S1 and S2 toward the minimum of S0 and decreases the energy barrier, favoring state transitions. Moreover, the power stroke step *d*(*x*_0_) and the total force decrease. One may wonder how the altered dynamics at the single cross-bridge level affect the macroscopic muscle contraction. To answer this question at the fiber level, we introduce the three scenarios (SRI, SRII, SL) into a single sarcomere model. Moreover, we assume, for simplicity, that all sarcomeres aligned in series and parallel within a single fiber behave in the same way; therefore, we can simulate the total tension generated by the fiber. We also simulate the rising phase of an isometric contraction and two experimental setups: the force clamp and the length step. The former experiment analyzes the constant velocity of contraction under different constant loads applied to the fiber; the latter analyzes the force recovery after a small, fast change in the fiber length. During an isometric contraction, the wider minima generate a higher force with fewer attached cross-bridges (Fig 3). This improved performance is attributable to the higher average population in the second minimum with respect to the total number of attached cross-bridges *S*_*TOT*_ in SL (43% of *S*_*TOT*_, see S1 Table) than in SRII (29%) and SRI (12%). The reduced number of attached cross-bridges is a result of the hypothesis of faster detachment from the more stretched elastic component. The wider minima also accelerate the attachment–detachment cycle by increasing the contraction velocity against a constant force. For any given force, SL achieves a higher contraction velocity than SRI and SRII (Fig 4). With the selected relative values of the parameters, the maximum velocity is comparable to the experimental data. However, the parameter values were identical in the three scenarios, so the faster velocity resulted solely from the altered energy landscape.

Let us assume that the half-sarcomere model is in mechanical equilibrium with the constant external force *F* pulling the Z-line. For simplicity, we assume that the myosin elastic element has linear elasticity with the force constant *k*. Then, we have:

$$k\sum_{i}\delta_{s}^{i}\left(t\right)\left[\left(x^{i}\left(t\right)-x_{0}^{i}\left(t\right)\right)+\left(x_{0}^{i}\left(t_{a}^{i}\right)-z\left(t\right)+z\left(t_{a}^{i}\right)\right)\right]=F$$ (8)

Here, the myosin proteins are indexed with *i*, and *δ*_*s*_ is equal to 1 if the myosin is attached and 0 otherwise. Let us estimate the increment Δ*z* given by the state transitions within the strongly attached state (changes of $x^{i}\left(t\right)-x_{0}^{i}\left(t\right)$) in the time interval [*t*: *t* + Δ*t*]. For simplicity, we assume that there are no transitions between the strongly attached state and the other states for any myosin. Then, we have:

$$k\sum_{i}\delta_{s}^{i}\left(t\right)\left(\Delta \left(x^{i}-x_{0}^{i}\right)-\Delta z\right)=0$$ (9)

where $\Delta (x^{i}-x_{0}^{i})$ is the strain increment given by the power strokes in [*t*: Δ*t*]. The above equation can be rewritten as

$$\frac{\Delta z}{\Delta t}=\frac{1}{\Delta t}\frac{\sum_{i}\delta_{s}^{i}\left(t\right)\Delta x^{i}}{\sum_{i}\delta_{s}^{i}\left(t\right)}=\frac{\Delta X_{PS}/\Delta t}{\sum_{i}\delta_{s}^{i}\left(t\right)}$$ (10)

where Δ*X*_*PS*_/Δ*t* is the generation rate of the power strokes in the hypothesis that no attachment or detachment occurs in [*t*: *t* + Δ*t*]. If we take into account the strain at the elastic part given by attachment and lost by detachment, we can rewrite the numerator in Eq (10) as (Δ*X*_*A*_ + Δ*X*_*PS*_ − Δ*X*_*D*_)/Δ*t*, where Δ*X*_*A*_ and Δ*X*_*D*_ are the magnitude of strain given by the attachments and lost by the detachments, respectively, in [*t*: *t* + Δ*t*]. The above equation means that the sliding velocity varies inversely with the number of attached myosin heads for the same generation rate of power strokes. This explains the superiority of SL for achieving faster shortening.

**Fig 5:**
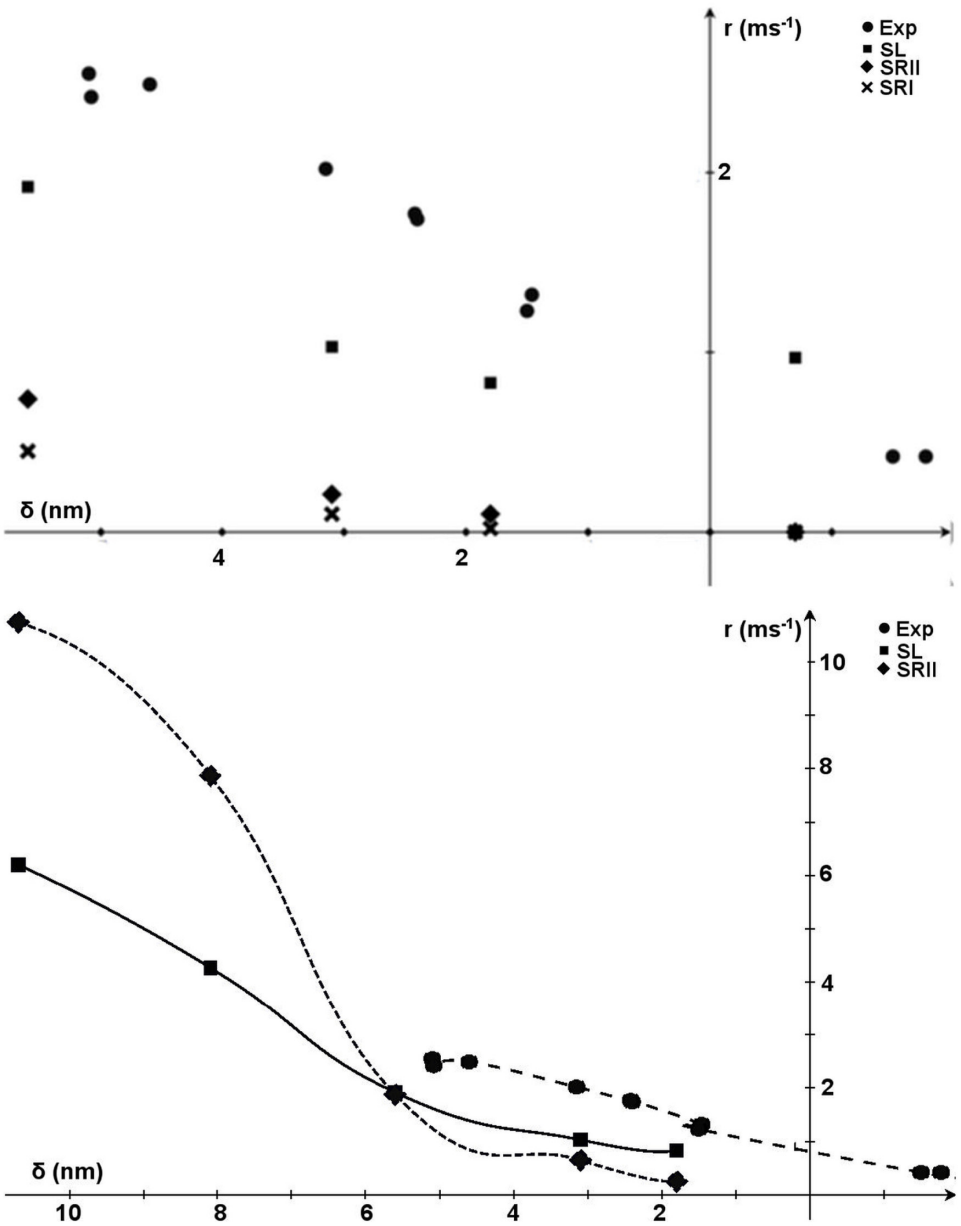
Rate of tension recovery after a small step of length *δ* before (upper panel) and after (lower panel) increasing the value of the free constant. Circles: experimental data, squares: SL, diamonds: SRII, crosses: SRI. The rate of recovery *r*(*δ*) is faster in SL than in SRII and SRI for the common parameters. Forcing the recovery rate for SRII to approach that of SL at *δ* = 5.6 *nm* results in overestimates and underestimates of the recovery rate itself at higher and lower values of *δ*, respectively. Experimental data are from [34] and the temperature was set to 4°C according to experimental results.

One might question whether the difference in myosin dynamics would persist after modifying the unloaded rate constants in SRI and SRII, which are here chosen to equalize that in SL. This modification would accelerate the transitions in the attached state, and also modify the fast recovery of the tension after a small length change. When an isometrically contracting muscle is subjected to a small, rapid length change, the tension almost instantaneously changes from its isometric value *T*_0_ to *T*_1_, then partially recovers to its original value, reaching a level *T*_2_ (see SI) at a rate *r* that reflects the power stroke velocity. *T*_1_, *T*_2_, and *r* depend on the length change *δ*. Fig 5 (upper panel) plots the tension recovery rate computed by exponentially fitting the tension vs. time curve. The recovery rate better matches the experimental results in SL than in the other scenarios with their chosen constants. Increasing the value of *k*_0_ in SRII (Eq (4)) such that the recovery rate approaches that of SL at *δ* = 5.6 *nm* results in overestimates and underestimates of the recovery rate itself at higher and lower values of *δ*, respectively (Fig 5, lower panel). This result shows that the choice of the constant parameter alone cannot justify the differences in the previous analysis. Moreover, the shape of the *r*(*δ*) curve supports the initial choice of *k*_0_.

### Heart

**Fig 6:**
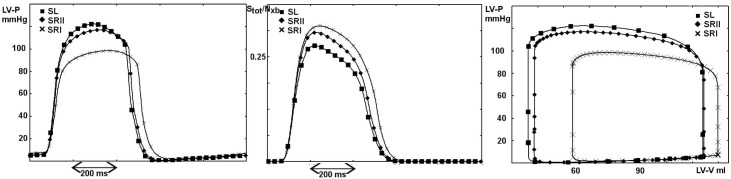
Beating heart analysis. Squares: SK approximation of SL, diamonds: SRII, crosses: SRI. Left ventricle pressure for the three scenarios: the SL case generates the highest peak pressure (left panel), despite having the lowest percentage of attached cross-bridges (central panel). SRI is the worst scenario. The temperature was set to 37°C. The three heart cycles are shown in the right panel.

**Fig 7:**
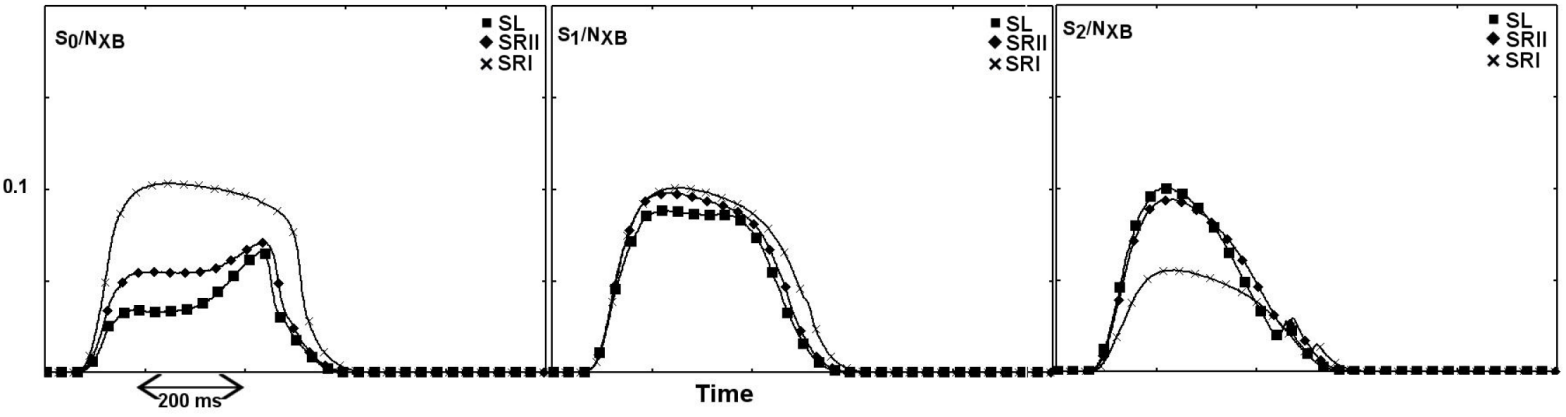
Populations of the three minima during one cardiac contraction cycle. The percentages reflect the behavior predicted at the single sarcomere level. The total number of attached cross-bridges is lower in SL (squares) than in SRI (crosses) and SRII (diamonds). However, the total pressure is higher in SL because the S2 state is more populated than in SRII and (especially) SRI. On the contrary, the S0 and (to a lesser extent) S1 states are more populated in SRI and SRII than in SL.

At the single-sarcomere level, the cross-bridges interact with each other through rigid actin and myosin filaments. In the whole fiber, the geometry is simplified by considering the sarcomeres to have uniform behavior. Whether these effects are preserved in a more physiological situation with several half-sarcomeres interacting in series and in a more complicated geometry is not a trivial question. To explore this idea further, we incorporated the three single-fiber models into a finite-element ventricle model. As shown in Fig 6, the previous differences are maintained at the macroscopic level of the heart. This result is non-trivial, because our analysis was made *ceteris paribus*, that is, we altered only the shape of the biochemical energy within and between the minima. All other parameters, such as the number of minima, the distance and energy drop between minima, constants of the attachment–detachment process, and geometric parameters, were unchanged. Importantly, thermal fluctuations inside the energy minima are usually neglected in mathematical models of muscle mechanics, which typically adopt the infinitely sharp minima hypothesis. Widening the minima increases the left ventricular pressure, leading to a higher ejection fraction. The relaxation is also accelerated, probably because of faster detachment (predicted at the single fiber level). The wider minima improve the general performance of the ventricle model, although SRII also generates physiological behavior. With the same parameter values, the SRI scenario shows limited ability to replicate a healthy heart. The higher pressure and ejection fraction in SL than in SRII are again linked to the lower percentage of attached cross-bridges. During the contracting phase, S2 is more populated in SL than in SRII. On the contrary, S0 and S1 are more populated in SR than in SL (Fig 6 (central panel) and Fig 7). It is worth noting that the simulated percentages of attached cross-bridges agree with the values inferred in [35]. In the simulations, only 20% of the simultaneously attached myosin heads generated the proper stroke volume. Finally, the contraction efficiency (defined in SI) is higher in the wider minima case of SL (28.7%) than in SRII (25.8%) and especially in SRI (16.1%). We quantitatively analyzed the dynamics of the cross-bridges during one heart beat in three different sarcomeres, placed at the endocardium, the epicardium, and between these two regions. Although the number of attachment and detachment events was quite similar in the three scenarios, the number of backward jumps was much higher in SL than in SRII and SRI. In SL, each attached cross-bridge made approximately five backward (followed by forward) jumps between the minima. In the sharp minima case, less than one-third of the cross-bridges demonstrated such dynamic behavior.

### Conclusions

By varying the shape of the minima and the intervening maxima of the potential energy of the actomyosin complex, this study explored the influence of the sharpness of the minima on the predicted actomyosin dynamics in three different mathematical models. We compared three scenarios, one corresponding to the classical Huxley and Simmons hypothesis [1], one following the analysis introduced in [24], and one introduced in [36]. The differences among the scenarios were compared at the single molecule, sarcomere, and whole-organ levels. Mathematically, the first two scenarios are characterized by infinitely sharp minima, whereas the third introduces a finite stiffness into the minima. In physical terms, the actomyosin complex in the first two scenarios is allowed to jump between stable states with rigid configurations, whereas the third scenario allows small thermal fluctuations within the stable configurations. In the vast majority of mathematical muscle models in the literature, the sharpness is effectively infinite because of the relatively simple expression of the detailed balance condition. Although it is now 40 years since the theoretical formalism that relates the rate constant between states to the mechanical and chemical energy shape was introduced [5], to the best of our knowledge, there has been no quantitative analysis of the consequences of the above-mentioned hypothesis. With no further differences in the actomyosin energy or the whole cross-bridge cycle, we showed that the shape of the minima in the energy landscape plays a fundamental role in the predicted dynamical behavior of each myosin molecule attached to an actin filament. In particular, widening the minima induces several rapid state transitions in single attachment–detachment events, whereas sharp minima generate few or single power stroke events. Moreover, allowing thermal fluctuations improves the predicted efficiency of the muscle contraction at all levels (single molecule, fiber, and whole organ).

In the consensus hypothesis, the myosin heads always attach in the pre-power stroke state. Consequently, they reach thermal equilibrium faster when rapid state transitions occur between the minima in the energy landscape. This dynamical behavior increases the populations of the force-generating states. The lower the lifetime of the actomyosin complex and the transition rates, the higher the percentage of time spent in the pre-power stroke (the low-force-generating state). In other words, because the cross-bridges always attach in the low-force state first, a faster state transition generates a higher probability of being in the high-force-generating state during the same actomyosin lifetime. As the values of the three energies in the minima are the same, this probability is the same in the steady state distribution, but the difference becomes negligible only at sufficiently long actomyosin lifetimes. Importantly, the shape of the biochemical component only has a non-trivial effect on the cross-bridge dynamics in relation to the mechanical components: wider minima reduce the effect of the mechanical energy, such that the minima in S1 and S2 have a lower energy barrier than in the infinitely sharp minima case, and shift toward the minimum in S0. The importance of this interaction is also revealed in the dependence of the rate constants on the stretching of the elastic component (S7 Fig). Comparing the forward rates *k*_01_ in SRII and the KS approximation of SL, we found that they substantially differ when the myosin is under high tension, but are similar when the tension is low or oppositely directed, as occurs in the step length protocols. As already mentioned in [24], single-molecule experiments may allow the testing of different energy shapes, through the exploration of observables under different setups.

As we have said, relaxing the infinitely steep minima hypothesis results in some fundamental differences from previous models. The attached myosin moves faster between stable states, the mean force generated per attached head is higher, and the heart simulator shows a faster drop in tension during relaxation. In principle, these effects can also be reproduced by other models, imposing different rate constants that are largely unknown at present. Despite this, the detailed balance condition on the ratio of backward and forward rate constants between stable states must be respected to ensure the consistency of any model [3]. We have already shown that the behavior predicted by the open minima model cannot be exactly reproduced by the steep minima models by simply modifying a single rate constant (Fig 5). Thus, other parameters in the model must be modified, such as the energy minima values or the elastic stiffness. In turn, this will modify other behavior simulated by the model, and eventually force the introduction of new, ad hoc hypotheses to overcome the new discrepancies. Obviously, this is only true if the real shape of the energy in muscles is relatively wide.

The influence of the wideness of the minima in the attached state has been presented in the framework of the lever arm hypothesis, currently the most widely accepted theory. However, to the best of our knowledge, the rotation of myosin molecules has not yet been observed. Indeed, the observed dynamical behavior of single myosin molecules may suggest a different mechanism [13], as has recently been supported by molecular dynamics simulations [37, 38]. In the new model, the myosin head thermally fluctuates on the actin filament, generating force until it reaches a strong-binding site (“hopping” assumption). Our wide minima approach can actually be applied to both mechanisms, with a different interpretation of the multi-stable biochemical energy. In the lever-arm assumption, different minima are associated with different stable states of the lever arm, where the myosin globular portion is always attached to the same actin monomer. The wideness of the minima is then related to the internal rigidity of the actomyosin complex in each stable state. In the hopping assumption, the potential arises from electrostatic interaction [37] between myosin and different actin monomers, generating different minima. The two assumptions would not affect the mathematical treatment. Notably, the apparent stiffness used in our definition of *E*_*c*_ around the minimum is about 48*pN*/*nm* (see Material and Methods), comparable with the stiffness within the minima obtained in [37] (about 16*pN*/*nm* at 25*mM* ionic strength, which is expected to increase at physiological ionic strengths). We have imposed an energy drop in *E*_*c*_ that requires almost all ATP energy (16*κ*_*b*_ *T*_0_), whereas in this previous study, the energy drop seems to be much lower. A complete comparison of the two models is beyond the scope of this paper.

All mathematical models of muscle mechanics are limited by the unknowability or wide variability of the real values of several parameters under physiological conditions. The present analysis is no exception. The influence of the energy landscape shape on myosin dynamics is probably affected by the choice of the attachment and detachment parameters and the common parameters of the actomyosin energy landscape. Moreover, although we have accounted for several components of the sarcomere and whole organs, other factors that have been neglected may influence the final behavior. For instance, we have not included the rigor states or the elasticity of the thin and thick actin filaments. Despite these omissions, the general behavior and time scales agree with several physiological and experimental data, demonstrating the robustness of the model with the chosen parameter set.

Importantly, the heart simulator integrated with the SL model exhibits a quantitative fit with several experimental observations of muscle contraction, from the single-molecule level to the whole heart. Rather than averaging, we considered the dynamics of each single myosin head. The model quantitatively matched the contraction velocity and the *T*_1_ and *T*_2_ curves, supporting the choice of the common parameters used. Moreover, as shown in Fig 4, the model reproduced the experimental observations of the four phases when transiting from an isometric to an isotonic contraction. Using such a model, we might elucidate the physiological meaning of several cardio-myopathies at the single-molecule level and connect them to whole-heart dysfunctionalities.

## Supporting Information

S1 Supporting Information Supporting Text. (PDF)

S1 Fig Schematic of the model, showing the TT units and the overlapping/overstretching mechanism. The lower part shows the SL (left) and the SR (right) representations of the attached state. Modified from [21].
(EPS)

S2 Fig Calcium transient. Imposed free [*Ca*^2+^] transient in time for the whole-heart simulations.
(EPS)

S3 Fig Comparison of the isotonic contraction in SL and its KS approximation for two values of external tension. The behavior of SRII is also reported for comparison. The SK approximation slightly overestimates the isotonic contraction at very low (zero) tensions.
(EPS)

S4 Fig Comparison of the length step protocol in SL and its KS approximation (*δ* = 12 *nm*). The behavior of SRII is also reported for comparison. The SK approximation fits the recovery curve well, but computes a slightly higher total tension than SL.
(EPS)

S5 Fig Definition of *T*_2_(*δ*) and *r*(*δ*). The population in S2 rapidly increases after the length change because of the power stroke of the old myosin. At a later time, it decreases because the myosin heads detach and the new myosin heads attach in S0. *T*_2_(*δ*) is defined as the tension at the time of the maximum in S2, and *r*(*δ*) as the inverse of the time required for the tension to reach *T* = 2/3*T*_2_.
(EPS)

S6 Fig Fast recovery in tension after a small step of length *δ*. Circles: experimental data, squares: SL, diamonds: SRII, crosses: SRI. The relative tension recovery *T*_2_/*T*_0_, computed as described in S5 Fig, is almost equal in all three scenarios, whereas the rate of recovery *r*(*δ*) (inset) is faster in SL than in SRII and SRI. Experimental data are from [18], and the temperature was set to 4°C according to experimental data.
(EPS)

S7 Fig Direct comparison of the rate constants computed in SRII and the KS approximation of SL. Widening the minima alters the probability of jumps among the attached stable states S0, S1, and S2. This effect is non-trivially dependent on the mechanical energy component. The rates change quite markedly when the elastic element is stretched, as during an isometric contraction, but are almost identical when the element is compressed, as in the length step protocol. This feature may also be important during some phases of cardiac contraction.
(EPS)

S1 Table Parameter values and their descriptions. (PDF)

S2 Table Attachment–detachment events. Counting of the Attachment–detachment events and backward jumps (BJ) (normalized by *N*_*XB*_) in the three scenarios, in three different regions of the left ventricle wall.
(PDF)
