# Supplementary material for: Including Thermal Fluctuations in Actomyosin Stable States Increases the Predicted Force per Motor and Macroscopic Efficiency in Muscle Modelling
Source: PLoS Comput Biol. 2016 Sep 14;12(9):e1005083. doi: 10.1371/journal.pcbi.1005083 (PMC5023195; doi:10.1371/journal.pcbi.1005083)
Supplement: S1 Supporting Information — (PDF) [file pcbi.1005083.s001.pdf]

Modeling Thermal Fluctuations in Actomyosin Stable States: An Overlooked Property in Muscle Models

Lorenzo Marcucci[1,2*◑], Takumi Washio[3,◑], Toshio Yanagida[2,◑],

1 Department of Biomedical Sciences, Padova University, Padova, Italy
2 Quantitative Biology Center, RIKEN, Suita, Japan
3 Graduate School of Frontier Sciences, The University of Tokyo, Tokyo, Japan

◑These authors contributed equally to this work.
*** lorenzo.marcucci@gmail.com**

S1 Supporting Information

S1.1 Model and numerical simulations

The myosin motor can exist in three states: detached (D), weakly attached (W), or strongly attached (S). The D and W states switch at constant transition rates k_{DW} and k_{WD}, respectively; the W–S (k_{WS}) and S–D (k_{SD}) transitions are functions of the myosin head position, as implemented in the Huxley 1957 model [1].

$$k_{WS} = \begin{cases} 0, & x < 0 \\ \alpha_a x, & 0 < x < l_t \\ 0, & x > 0 \end{cases}$$

$$k_{SD} = \begin{cases} D_n, & x < 0 \\ \alpha_d x, & x > 0 \end{cases}$$

The S state is described as a continuous energy landscape with three minima, corresponding to a pre-power stroke (S0) and a power stroke with two stages (S1 and S2). In SL, the central region of the biochemical energy landscape is defined as in Equation 5. ATP energy release favors the power stroke by biasing the biochemical energy such that $E_c^{S0} > E_c^{S1} > E_c^{S2}$. To simulate this bias, we add a linear drop F_{atp} to the flat sinusoidal part of E_c. α_d is a constant angle that adjusts for the constant ATP drop by shifting the first minimum to zero. The convex part is expressed by a polynomial, ensuring a continuous first derivative. The energy barrier in the sinusoidal function is chosen as $H = 6\kappa_b T_0$ (in the two flat landscapes, the maximum is less important because it is included in the unloaded rate constants; see main text). We can then approximate the minimum in E_c as a parabola with stiffness $H(2\pi/d)^2$, which corresponds to $k_c = 48\ pN/nm$. Therefore, the energy bias due to the ATP is $F_{atp} = 8\kappa_b T_0/d$ (where $T_0 = 37\,°\mathrm{C}$ is the reference temperature and d is the distance between two consecutive minima). Note that the total energy drop due to the ATP in the biochemical component is $16\kappa_b T_0$, below the total amount of energy released by ATP hydrolysis (approximately $18\kappa_b T_0$). The ATP energy difference is here attributed to Huxley's 1957 [1] hypothesis, whereby preferential attachment at higher stretching levels requires energy to break the detailed balance. According to H57, the pre-power stroke state also generates a force, here justified by the above-mentioned ATP energy difference.

S1.2 Number of myosin molecules, TT units, Cooperativity and Geometry

The lengths of the half-actin and myosin filaments are 1224 nm and 925 nm, respectively. The myosin filament includes 50 nm of bare zone with no myosin heads, implying 60 myosin molecules per thick filament. Each thick filament is surrounded by six double-helix thin filaments, each of which can be reached by the myosin heads on three thick filaments; therefore, each myosin filament contains approximately 30 double-headed myosin molecules, which can interact with each actin filament. Because troponin–tropomyosin (TT) units are related to each single strand in the helix, this number is strictly halved, but is restored to 30 to accommodate two myosin heads per molecule. To allow for possible interaction from other myosin heads [3], we increase the number of myosin heads interacting with each actin filament to 38, as in [2]. The total force is then multiplied by two to recover the double-stranded helix.

Muscle contraction and release is governed by the amount of Ca in the troponin binding sites, which is able to shift the tropomyosin filament exposing F-actin to myosin motors. The occupancy of these binding sites is governed by the myoplasmic free $[Ca^{2+}]$, which diffuses, following an action potential on the cell membrane, from the sarcoplasmatic reticulum (SR) release channels to the myofibrillar space, and is subsequently taken up by the SR Ca pumps. As a consequence, the myoplasmic free $[Ca^{2+}]$ depends on the particular experimental or physiological situation. The actin filament houses several TT units, each with two Ca affinity sites, spaced at $36nm$ intervals. The rate of Ca ion occupancy depends on the instantaneous Ca concentration. When both Ca sites are empty (Ca-off in S1 Fig) or only one site is filled (Ca-on*), the TT unit is inactive and the corresponding portion of the actin filament has lower affinity for the myosin head (modeled through the variable Q_{Ca}). The affinity increases when both sites are filled (Ca-on). Moreover, if at least one myosin is already attached to an actin monomer corresponding to a TT unit, the deformation of the tropomyosin filament reduces the detachment rate of the captured Ca ions (by factors of 4 and 2 in Ca-on $\rightarrow$ Ca-on* and Ca-on* $\rightarrow$ Ca-off transitions, respectively).

In single-fiber simulations, we impose a tetanic state such that the myoplasmic free $[Ca^{2+}]$ is constant and sufficiently high to saturate the Ca binding sites in the troponin. In the whole-heart simulations, the free $[Ca^{2+}]$ transient is imposed as in S2 Fig In the whole-heart simulations, the free Ca at each side of the ventricle is computed by conducting an excitation propagation simulation [4], whereby the human cardiomyocyte model [5] is applied for the computation of the ion channel currents. S2 Fig shows the time evolution of the Ca transient averaged over the ventricle.

In the single-fiber simulations, the calcium concentration is fixed at its saturated value (tetanic situation), whereas in the whole-heart simulation, the Ca concentration follows a realistic time-transient trend. Moreover, the transient is shifted in each sarcomere, approximating the real spreading of an electrical signal.

The cooperativity of myosin attachment affects the transition rates k_{DW} and k_{WD} through a parameter γ^n, where n is 0 if there are no nearest neighbors and 1 or 2 if one or two myosin heads are already weakly or strongly attached, respectively. Finally, to account for the overlapping of filaments in over-compressed or over-stretched situations, we multiply k_{WS} by a sigmoidal factor χ:

$$\chi = \begin{cases} 1, & x_s < 0 \\ \exp(-x_s^2/a_s^2), & x_s > 0 \end{cases}$$

where x_s is the overlapping or overstretched distance of the given myosin head and a_s is a constant. In the absence of overlapping or overstretching, $x_s < 0$. These three effects

are modeled as described in [2], which gives:

$$k_{DW} = k_{DW}^0 Q_{Ca} \gamma^n \chi Q_{10}$$

At each time step, the algorithm updates the position x_i of the $i-th$ myosin head with respect to its anchor on the thick filament. In this way, the total tension generated is given by:

$$F_{Tot} = 2 \sum_{j=0}^{N_{fil}} \sum_{i=0}^{N_{XB}} k(x_i) x_i \quad \text{(S1)}$$

where N_{XB} is the total number of myosin heads, N_{fil} is the number of filaments in the sarcomere, and $k(x_i)$ explicitly expresses the dependence of the asymmetric stiffness on the myosin position. The factor of two accounts for the second strand, as mentioned above. In SRI and SRII, the position of the myosin head is first updated based on the position of the actin filament, z, in the previous time step:

$$x_i(t) = z(t) - z_i^a + x_i^a + n_{ps} d \quad \text{(S2)}$$

Here, z_i^a and x_i^a are the positions of the actin filament and the myosin pre-stretch, respectively, at the time of attachment of the $i-th$ myosin head. n_{ps} equals 0, 1, or 2 when the myosin head resides in the S0, S1, or S2 minimum, respectively. Having updated the position of each myosin head, the algorithm updates the position of the actin filament using Newton–Raphson method. In each iteration, the total force is computed by Equation S1.

In the implicit scheme, an equation of the form

$$q(\dot{u}, u) + f = 0 \quad \text{(S3)}$$

is approximately solved so that the following two equations are simultaneously satisfied at every time step from t to $t + \Delta t$.

$$q({}^{t+\Delta t}\dot{u}, {}^{t+\Delta t}u) + {}^{t+\Delta t}f = 0 \quad \text{(S4)}$$

$${}^{t+\Delta t}u = {}^{t}u + \Delta t \left[\bar{\gamma}\ {}^{t+\Delta t}\dot{u} + (1-\bar{\gamma})\ {}^{t}\dot{u}\right] \quad \text{(S5)}$$

where q is the internal force vector given as a function of $\dot{u}$ and u, and f corresponds to the external force vector. In the single-fiber simulations, u is an $(N_x b * N_f il + 1)$ vector of the positions of the myosin motors and actin filaments, whereas $\dot{u}$ represents their velocities. q is the force generated by myosin elastic elements and their drag force, as well as the drag force on the actin filament. f is the external force applied to the actin filament (when present). In SL, this also contains the random forces acting on each myosin motor. Finally, $\bar{\gamma}$ is a parameter of between 0 and 1. In our case, $\bar{\gamma} = 0.6$. In the heart simulator, we use a similar but more complicated procedure, because we apply a number of Monte Carlo time steps ($\Delta t = 1\ \mu s$) within each Finite Element Model time step ($\Delta t = 1.25\ ms$). Refer to [2] for a detailed explanation.

In Newton-Raphson method, Equations S4 and S5 are iteratively solved by linearizing the internal force term, as described in the following procedure. First, the initial guess is given by:

$${}^{t+\Delta t}\dot{u}^{(0)} = {}^{t}\dot{u} \quad \text{(S6)}$$

$${}^{t+\Delta t}u^{(0)} = {}^{t}u \quad \text{(S7)}$$

Then, the first iteration is conducted as follows:

Residual computation:

$$r^{(0)} = {}^{t+\Delta t}q({}^{t+\Delta t}\dot{u}^{(0)}, {}^{t+\Delta t}u^{(0)}) - {}^{t+\Delta t}f \quad \text{(S8)}$$

Solution for the velocity update:

$$(C^{(0)} + \bar{\gamma}\Delta t K^{(0)})\Delta\dot{u}^{(1)} = r^{(0)} - \Delta C^{(0)t}\dot{u} \tag{S9}$$

Updating:

$$^{t+\Delta t}u^{(1)} = {}^{t+\Delta t}u^{(0)} + \bar{\gamma}\Delta t\Delta\dot{u}^{(1)} + \Delta t^{t}\dot{u} \tag{S10}$$

$$^{t+\Delta t}\dot{u}^{(1)} = {}^{t+\Delta t}\dot{u}^{(0)} + \Delta\dot{u}^{(1)} \tag{S11}$$

Here, $C^{(0)} = \frac{dq}{d\dot{u}}({}^{t+\Delta t}\dot{u}^{(0)}, {}^{t+\Delta t}u^{(0)})$, and $K^{(0)} = \frac{dq}{du}({}^{t+\Delta t}\dot{u}^{(0)}, {}^{t+\Delta t}u^{(0)})$.

From the second iteration ($k > 0$), the following procedure is conducted until the residual norm becomes sufficiently small.

Residual computation:

$$r^{(k+1)} = {}^{t+\Delta t}q({}^{t+\Delta t}\dot{u}^{(k)}, {}^{t+\Delta t}u^{(k)}) - {}^{t+\Delta t}f \tag{S12}$$

Solution for the velocity update:

$$(C(k) + \bar{\gamma}\Delta t K(k))\Delta\dot{u}^{(k+1)} = r^{(k+1)} \tag{S13}$$

Updating:

$$^{t+\Delta t}u^{(k+1)} = {}^{t+\Delta t}u^{(k)} + \bar{\gamma}\Delta\dot{u}^{(k+1)} \tag{S14}$$

$$^{t+\Delta t}\dot{u}^{(k+1)} = {}^{t+\Delta t}\dot{u}^{(k)} + \Delta\dot{u}^{(k+1)} \tag{S15}$$

Here, $C^{(k)} = \frac{dq}{d\dot{u}}({}^{t+\Delta t}\dot{u}^{(k)}, {}^{t+\Delta t}u^{(k)})$, and $K^{(k)} = \frac{dq}{du}({}^{t+\Delta t}\dot{u}^{(k)}, {}^{t+\Delta t}u^{(k)})$.

The difference between the first and subsequent iterations in the update of u is necessary to satisfy Equation S5. This generates the difference in the right-hand side of the solution for the velocity update. In SL, Equation S2 is not used; rather, the actomyosin complex is treated as a diffusing, over-damped particle with drag coefficient η. The complex is subjected to thermal, elastic, and chemical energies, the latter describing the actomyosin interaction and the ATP effect. In the equation in S1 Fig, the biochemical and elastic (mechanical) components are separated as E_c and E_e, respectively, and the third term describes the temperature effects. $\omega = 0$ when the myosin head is detached and 1 when it is attached. $\Gamma(t)$ is a random term satisfying $< \Gamma(t) >= 0$ and $< \Gamma(t_1), \Gamma(t_2) >= 2\delta(t_1 - t_2)$ (white noise). The total tension is computed as:

$$T_{tot} = F_{tot}/A_f \tag{S16}$$

where A_f is the cross-sectional area of one filament.

The work done by each ventricle is computed as the ejection flux multiplied by the pressure at the valves minus the work done by injection. The efficiency of the whole heart is computed as the ratio of total work to total ATP consumption.

At each time step Δt and for each possible transition, a random number, uniformly distributed between zero and one, is generated. The transition occurs if this value is less than or equal to Δtk, where k is the defined rate constant. $\Delta t = 1\ \mu s$ in SRI, SRII, and the KS approximation of SL, and $\Delta t = 1\ ns$ in SL. The value of k is modified accordingly. At the single cross-bridge and single sarcomere/fiber levels, the numerical simulations of scenarios SRI and SRII and scenario SL differ as follows.

The algorithm for SRI and SRII is:

1. Generate a matrix of rate constants for each stretching level between $-20\ nm$ and $20\ nm$. The rate constants are given by Equation 4 and by trapezoidal integration of Equation 3 for SRII and SRI, respectively (see main text).

2. Update the states of the TT units depending on the instantaneous Ca concentration.

3. Define the stable state of each myosin head (D, W, S0, S1, or S2) using the rate constants k_{DW}, k_{WD}, k_{WS}, k_{SD} or the rate constants produced in step 1 (k_{12}, k_{21}, k_{01}, k_{10}), depending on the stretching of the elastic component of the myosin head.

4. Update the positions of each myosin head and actin filament, depending on the setup and external conditions, and calculate the total force.

5. Increment the time step and return to Step 2 until the total time is equal to the prescribed simulation time.

The algorithm for SL is slightly different:

1. Update the states of the TT units depending on the Ca concentration.

2. Define the stable state of each myosin head (D, W, S) using the rate constants k_{DW}, k_{WD}, k_{WS}, and k_{SD}.

3. Using an implicit method, determine the position of each myosin head from Equation 6.

4. Update the external conditions depending on the setup and calculate the total force in the current time step.

5. Increment the time step and return to Step 1. Repeat until the total time is equal to the prescribed simulation time.

To account for the different temperatures in the experimental observations of single fibers and cardiac contraction, we include a factor Q_{10} in the definitions of k_{DW}, k_{WD}, k_{WS}, and k_{SD}. We assume that the biochemical and mechanical energy components are independent of temperature. The temperature effects on the oscillations are introduced through $\kappa_b T$ in the rate constants of SRI and SRII and in the Langevin equation for SL. The temperature effect on the TT units is neglected. The Langevin approach is unsuitable for multiscale analysis because of its intrinsic time step limitations. Instead, we use the KS approximation of the SL. The algorithm is the same as that used for SRII, but the rate constant matrix is generated by the KS approximation of the Langevin equation (see Equation 6). To account for the oscillations within the minima (and in the detached and weakly attached states), the position of each myosin head at each time step is computed by randomly selecting a position from the corresponding stationary probability distribution in the current state:

$$p_s(x) = N \exp(G(x, x_0)/\kappa_b T)$$

where N is the normalization constant. This action modifies the last term in Equation S2 to account for the shifting of the total energy minima under the wide minimum hypothesis.

S1.3 Comparison between the Langevin and Kramers–Smoluchowski (KS) approximations

The numerical approximation to the system of Langevin equations describing the motion of each myosin molecule has several advantages, as it reproduces the detailed myosin dynamics, including the real effect of the energy barrier shape on the dwell time of the protein and other phenomena that cannot be simulated by different approaches. However, its applicability is limited by the long computation time, because the time step for the analysis must be smaller than the typical timescale of the process. In the

myosin analysis, the time step is a few nanoseconds, which is feasible for simulations of single sarcomeres, but not for whole-heart simulations. For this reason, we adopt a rate constant approach based on the KS approximation of the energy landscape used in SL at the single sarcomere level. Here, we show that the approximation is sufficient for the purpose of our study by comparing the results of the KS approximation with the force clamp and the length step protocols in both SL and SRII. As shown in S3 Fig and S4 Fig, the approximation follows the general behavior of the SL model, and remains clearly distinct from the SRII results. In the isokinetic contraction, the agreement is particularly strong at intermediate levels of tension (S3 Fig).

At low tensions, the KS approximation slightly overestimates the contraction velocity and the total tension. However, the recovery velocity after a small length change is similar to the SL case, as shown in S4 Fig

S2 Tension recovery after a small step

When a small, fast length change is imposed on an isometrically contracting muscle, the tension changes almost instantaneously from T_0 to T_1, then exponentially recovers toward T_0 with a rate r, settling at a new value T_2. The initial change is related to the mechanical relaxation of the elastic element, and the recovery is related to the equilibrium change caused by the power stroke [6]. r, T_1, and T_2 depend on the imposed step δ. As experimentally observed, the T_2 value is somehow hidden by the detachment of old myosin heads and the attachment of new motors. To overcome this problem, we consider the population of cross-bridges in S2 to reach a maximum when the old cross-bridges have completed the power stroke. For this reason, we define the values of the variable $T_2(\delta)$ and $r(\delta)$ at the time of the maximum in S2 (see S5 Fig).

The simulated data for T_1 and T_2 (see S6 Fig) are very similar in the three scenarios, and are comparable to the experimental behavior.

Acknowledgments

LM was partially supported by High Performance Computing Infrastructure (HPCI) Field 1 Supercomputational Life Science (Strategic institution: RIKEN) and European Commission, Seventh Framework Programme (FP7/2007-2013) under Grant Agreement no.600376. TW work was supported in part by MEXT as Strategic Programs for Innovative Research Field 1 Supercomputational Life Science and a social and scientific priority issue (Integrated computational life science to support personalized and preventive medicine) to be tackled by using post-K computer.

Table S1. Parameter values and their descriptions

Parameter	Value	Description
k	2-0.4 pN/nm	myosin stiffness
η	70 pNns/nm	myosin drag coefficient
N_{XB}	38	myosin per filament
N_{fil}	240-12	number of actin filaments (sarcomere - heart)
LB	50 nm	bare zone
LM	825 nm	myosin filament length
LA	1224 nm	actin filament length
d_{TT}	36 nm	distance between two TT units
Q_{Ca}	55	inhibiting parameter in absence of Ca
DT	1 μs	time step in single-sarcomere simulations
T	$4-37\,^{\circ}$C	temperature in single-sarcomere heart simulation
κ_b	0.0138 pN nm/K	Boltzmann constant
$X_t r$	10 nm	threshold distance for zero attachment rate
k^0_{WD}	$20\gamma^2 s^{-1}$	basic W to D rate
k^0_{DW}	72 s^{-1}	basic D to W rate
α_a	9 $s^{-1}nm^{-1}$	stretch dependence of W to S rate
α_d	$0.54\alpha_a$	stretch dependence of S to D rate
D_N	50 s^{-1}	S to D rate in compression
a_s	10 nm	factor of overlapping and overstretching
$Ca^*_{on} = Ca_{on}$	4800 $s^{-1}\mu M^{-1}$	attachment Ca rate on TT unit
$Ca^*_{off} = Ca_{off}$	9600 s^{-1}	detachment Ca rate on TT unit
A_f	10^{-3} μ^2	cross-sectional area per filament

Table S2. Attachment–detachment events and backward jumps (BJ) (normalized by N_{XB}) in the three scenarios, in three different regions of the left ventricle wall.

Events/ B J	Endo	Middle	Epi
SRI	11.2/0.75	10.5/0.6	7.8/0.3
SRII	10.0/3.2	9.5/2.2	6.11/1.5
SL	10.5/63.2	9.2/47.2	5.3/26.7

References

1. Huxley AF. Muscle structure and theories of contraction. Prog Biophys Biophys Chem. 1957;7:255–318.

2. Washio T, Yoneda K, Okada J, Kariya T, Sugiura S, Hisada T. Ventricular fiber optimization utilizing the branching structure. Int J Numer Method Biomed Eng. 2016 Jul;32(7):e02753.

3. Spudich JA. Hypertrophic and dilated cardiomyopathy: four decades of basic research on muscle lead to potential therapeutic approaches to these devastating genetic diseases. Biophys J. 2014 Mar;106(6):1236–1249.

4. Okada J, Washio T, Maehara A, Momomura S, Sugiura S, Hisada T. Transmural and apicobasal gradients in repolarization contribute to T-wave genesis in human surface ECG. Am J Physiol Heart Circ Physiol. 2011 Jul;301(1):H200–208.

5. ten Tusscher KH, Noble D, Noble PJ, Panfilov AV. A model for human ventricular tissue. Am J Physiol Heart Circ Physiol. 2004 Apr;286(4):H1573–1589.

6. Huxley AF, Simmons RM. Proposed mechanism of force generation in striated muscle. Nature. 1971 Oct;233(5321):533–538.

7. Piazzesi G, Lombardi V. A cross-bridge model that is able to explain mechanical and energetic properties of shortening muscle. Biophys J. 1995 May;68(5):1966–1979.
