# Supplementary material for: Including Thermal Fluctuations in Actomyosin Stable States Increases the Predicted Force per Motor and Macroscopic Efficiency in Muscle Modelling
Source: PLoS Comput Biol. 2016 Sep 14;12(9):e1005083. doi: 10.1371/journal.pcbi.1005083 (PMC5023195; doi:10.1371/journal.pcbi.1005083)
Supplement: S1 Table — (PDF) [file pcbi.1005083.s009.pdf]

Modeling Thermal Fluctuations in Actomyosin Stable States: An Overlooked Property in Muscle Models

Lorenzo Marcucci[1,2*◑], Takumi Washio[3,◑], Toshio Yanagida[2,◑],

1 Department of Biomedical Sciences, Padova University, Padova, Italy
2 Quantitative Biology Center, RIKEN, Suita, Japan
3 Graduate School of Frontier Sciences, The University of Tokyo, Tokyo, Japan

◑These authors contributed equally to this work.
*** lorenzo.marcucci@gmail.com**

Table S1. Parameter values and their descriptions

Parameter	Value	Description
k	2-0.4 pN/nm	myosin stiffness
η	70 pNns/nm	myosin drag coefficient
N_{XB}	38	myosin per filament
N_{fil}	240-12	number of actin filaments (sarcomere - heart)
LB	50 nm	bare zone
LM	825 nm	myosin filament length
LA	1224 nm	actin filament length
d_{TT}	36 nm	distance between two TT units
Q_{Ca}	55	inhibiting parameter in absence of Ca
DT	1 μs	time step in single-sarcomere simulations
T	$4 - 37\,^{\circ}$C	temperature in single-sarcomere heart simulation
κ_b	0.0138 pN nm/K	Boltzmann constant
$X_t r$	10 nm	threshold distance for zero attachment rate
k^0_{WD}	$20\gamma^2 s^{-1}$	basic W to D rate
k^0_{DW}	72 s^{-1}	basic D to W rate
α_a	9 $s^{-1}nm^{-1}$	stretch dependence of W to S rate
α_d	$0.54\alpha_a$	stretch dependence of S to D rate
D_N	50 s^{-1}	S to D rate in compression
a_s	10 nm	factor of overlapping and overstretching
$Ca^*_{on} = Ca_{on}$	4800 $s^{-1}\mu M^{-1}$	attachment Ca rate on TT unit
$Ca^*_{off} = Ca_{off}$	9600 s^{-1}	detachment Ca rate on TT unit
A_f	10^{-3} μ^2	cross-sectional area per filament
