# Supplementary material for: Including Thermal Fluctuations in Actomyosin Stable States Increases the Predicted Force per Motor and Macroscopic Efficiency in Muscle Modelling
Source: PLoS Comput Biol. 2016 Sep 14;12(9):e1005083. doi: 10.1371/journal.pcbi.1005083 (PMC5023195; doi:10.1371/journal.pcbi.1005083)
Supplement: S2 Table — Counting of the Attachment–detachment events and backward jumps (BJ) (normalized by NXB) in the three scenarios, in three different regions of the left ventricle wall. (PDF) [file pcbi.1005083.s010.pdf]

Modeling Thermal Fluctuations in Actomyosin Stable States: An Overlooked Property in Muscle Models

Lorenzo Marcucci[1,2*◕], Takumi Washio[3,◕], Toshio Yanagida[2,◕],

1 Department of Biomedical Sciences, Padova University, Padova, Italy
2 Quantitative Biology Center, RIKEN, Suita, Japan
3 Graduate School of Frontier Sciences, The University of Tokyo, Tokyo, Japan

◕These authors contributed equally to this work.
*** lorenzo.marcucci@gmail.com**

Table S2. Attachment–detachment events and backward jumps (BJ) (normalized by N_{XB}) in the three scenarios, in three different regions of the left ventricle wall.

Events/ B J	Endo	Middle	Epi
SRI	11.2/0.75	10.5/0.6	7.8/0.3
SRII	10.0/3.2	9.5/2.2	6.11/1.5
SL	10.5/63.2	9.2/47.2	5.3/26.7
